# Announcement: Therapeutic Uses of Trace Elements

**DOI:** 10.1155/MBD.1995.193

**Published:** 1995


					THERAPEUTIC USES OF
TRACE ELEMENTS
Satellite Workshop:
Molecular Basis for Copper Metabolic Disorders
Meribel (France)
February 4th-7th, 1996
5th International Congress on
Trace Elements in Medicine and Biology
Scientific Committee
Berthon G. (France)
Sorensen J. R. J. (USA)
Chappuis P. (France)
Favier A (France)
Milanino R. (Italy)
Marie P. (France)
Hercberg S. (France)
Local organizer
Boivin G. (France)
Chelly J.(U.K)
Haguenau M. (France)
Neve J. (Belgium)
Walvarens P. (USA)
Lamand M. (France)
Sturniolo G.C (Italy)
SFERETE, Soci6t6 Franaise d'Etude et de Recherche sur les EI6ments Traces
Official languages
English French (simultaneous translation)
Topics
Therapeutic Forms of Trace Elements
Presentaton, Dose, Chemical forms, Bioavailability and efficacy criteria, Mineral and vitamin associations
Trace Elements, bone physiology, bone diseases and rheumatic diseases
Fluoride, Zinc, Strontium, Silicon, Gallium
Trace Element status in relation with inflammatory conditions and infections
Treatment with trace elements, Immuno-modulation, Liver cirrhosis and pancreatitis, Dermatological diseases,
AIDS
Trace Elements and endocrinology
Thyroid hormones (selenium-iodine interactions), glucose intolerance, gonadal dysfunction
Pharmacological applications of Trace Elements Chrysotherapy, Fluoride and dental caries, Cancer
chemotherapy, Lithium therapy, Chelation therapy
Large epidemiological and intervention studies related to Trace Elements
Relation with and prevention of diseases such as cancer, Cardiavascular diseases, Degenerative disorders
Trace Elements supplementation of population groups at different period of age
Concelpts and effects in neonates, children, pregnant and lactating women, elderly, parenteral and enteral
nutrition, improvement of physical performances
Submission of Posters or oral communications
Additionnal information for the submission of abstracts will be given in the second announcement.
Abstracts
Abstracts of all the presentations including accepted papers will be distributed at the opening of the congress
to all registered participants and will be included in the registration fee.
Provisional time schedule
April 1995: 2nd announcement and registrati.on
October I 1995: Deadline for submitting abstracts
November I 1995: Authors will be informed of acceptance of abstracts.
For more information, please contact
Arlette ALCARAZ
Laboratoire de Biochimie C, C.H.R.U. de Grenoble, B.P. 217, F 38043 Grenoble, France
Tel.: (33) 76.76.54.84; Fax.: (33) 76.76.56.64
193

				

